# High-altitude environments enhance the ability of *Eothenomys miletus* to regulate body mass during food limitation, with a focus on gut microorganisms and physiological markers

**DOI:** 10.3389/fmicb.2024.1499028

**Published:** 2024-11-01

**Authors:** Tianxin Zhang, Ting Jia, Wanlong Zhu, Lixian Fan

**Affiliations:** ^1^Key Laboratory of Ecological Adaptive Evolution and Conservation on Animals-Plants in Southwest Mountain Ecosystem of Yunnan Province Higher Institutes College, Yunnan Normal University, Kunming, China; ^2^School of Life Sciences, Yunnan Normal University, Kunming, China; ^3^Key Laboratory of Yunnan Province for Biomass Energy and Environment Biotechnology, Yunnan Normal University, Kunming, China

**Keywords:** *Eothenomys miletus*, high altitude, food restriction, gut microbiota, adaptation

## Abstract

Animals’ digestion, energy metabolism, and immunity are significantly influenced by interactions between the gut microbiota and the intestinal environment of the host. Previous studies have shown that gut microbiota of *Eothenomys miletus* can respond to environmental changes, high fiber or fat foods. But how *E. miletus* in high-altitude adapt to their environment through gut microbiota and physiological changes during winter food shortages period was unclear. In the present study, we evaluated the altitude differences in gut microbiota and their interactions with physiology in terms of body mass regulation in order to study the adaptation of the gut microbiota and physiological indicators of the *E. miletus* under food restriction settings. *E. miletus* were collected for this study from Jingdong County (JD, low-altitude) and Xianggelila County (XGLL, high-altitude) in Yunnan Province, China, and split into three groups: control group, food-restricted feeding group for 7 days, and re-feeding group was offered a standard diet for 14 days. 16S rRNA gene sequencing and physiological methods were used to analyze the abundance and community structure of gut microbiota, as well as physiological indicators of each group in *E. miletus*. The results showed that while the RMR changed more during the period of food restriction, the body mass and major organ masses of *E. miletus* from high-altitude changed less. After food restriction, RMR in XGLL decreased by 25.25%, while that of in JD decreased by 16.54%. *E. miletus* from the XGLL had gut bacteria that were more abundant in Firmicutes and had fewer OTUs, and the microbiota had a closer interaction with physiological indicators. Moreover, the gut microbiota adapted to the food shortage environment by enhancing the genera of *Bacterroides*, *Ruminococcus*, *Turicibacter*, and *Treponema* to improve the utilization of nutrient resources. The interactions between microbial species and the equilibrium of energy homeostasis were further impacted by alterations in physiological indicators and microbial community structure. These variations were important for *E. miletus* to adapt to the fluctuations and changes of food resources in high-altitude region, which also expand our knowledge of organismal adaptations and the mechanisms behind the interactions between gut bacteria and host physiology.

## 1 Introduction

Animal host’s gut often contains a range of distinct bacterial communities, each of which serves an essential purpose (Koch et al., 2012). Interaction between host’s intestinal environment and the gut microbiota influence their thermogenesis, immunity, and metabolism (Zhou et al., 2023). The gut community may alter as a result of high-altitude environmental factors (Wang et al., 2022). For example, mammals and humans who were exposed to high altitudes for extended periods showed a higher proportion and relative abundance of Firmicutes and Bacteroidetes (Geng et al., 2023). By comparing fecal samples from mice living at high and low altitudes, it discovered substantial differences in the number of genera linked with inflammation, gastrointestinal illnesses in the gut microbiota of individuals from high altitudes (Zhang et al., 2018). There are also studies that showed spatial–temporal change in gut microbial function was more profound in the low-altitude macaques than in the high-altitude population (Li et al., 2023).

Selection pressures in high-altitude environments drive adaptations in organismal phenotypes (Hao et al., 2019). According to studies, yaks’ hearts and lungs in high-altitude were comparatively larger than those of their close relatives who reside at low-altitude (Guan et al., 2017). Bactrian camels living in higher altitude had a faster respiratory rate and lower blood glucose levels (Lamo et al., 2020), and plateau pikas living at high altitude had a higher resting metabolic rate (RMR) relative to individuals living at low altitude (Zhu et al., 2022). These results suggested that the animals had evolved a special physiological adaptation strategy for living at high altitudes for an extended period of time.

Food amount affects the diversity and community structure of an animal’s gut microbiota, and different animals’ gut microbiota experienced distinct alterations in response to food constraint (Li et al., 2024; Zha et al., 2018). Research showed that food restriction can alter the abundance of *Staphylococcus*, *Aerococcus,* and *Jeotgalicoccus* in Brandt’s voles (Dai et al., 2023). Furthermore, food restriction significantly altered the physiological characteristics of rodents. Rodents under conditions of food restriction exhibited reducing of energy expenditure, and changed metabolic processes of carbohydrates, proteins or lipids, along with corresponding enzyme activity (Liang and Zhang, 2003). Body mass of *Phodopus sungorus* and other hamsters dropped dramatically when food was scarce. However, upon refeeding, both body mass and body fat returned to the control level, demonstrating obvious plasticity (Keen-Rhinehart and Bartness, 2008; Del Valle et al., 2006). Energy metabolism of *E. miletus* was also influenced by food resource changes. Food affected the it’s regulation to adapt to different survival situations by controlling its internal organ body mass, digestive tract shape, and thermogenic capability (Zhu et al., 2009; Zhu et al., 2011; Mu et al., 2014).

The Hengduan Mountains, a unique alpine valley region in China that is at the intersection of the Eastern Oceanic and Palaearctic zones, are one of the world’s hotspots for biodiversity (Gong et al., 2001). Significant height disparities, temperature fluctuations, evident seasonal changes in vegetation availability, and discernible differences in the physiological and ecological traits of small mammals at various elevations are all present (Zhu et al., 2010). *E. miletus* belongs to the genus *Eothenomys*, Rodentia, which is endemic to China and inherent to the Hengduan Mountains (Chen et al., 2022). It generally eats carbohydrate and sucrose and feeds on the fresh sap of plants and grass roots (Gong et al., 2021). Mammals’ gut microbiota composition and functional selection were strongly impacted by altitude. For instance, the diversity of gut microbiota in high-altitude habitats has changed significantly in animals like wild sable, pika, and rhesus macaques (Su et al., 2021; Li et al., 2018; Wu et al., 2020). According to our previous researches, alterations in the structure and diversity of the gut bacterial community in *E. miletus* favorably correlated with environmental changes (Yan et al., 2022). Furthermore, changes in gut community composition and diversity under high-fiber diets can provide a significant safeguard for adapting in winter (Zhang et al., 2023). However, it is unclear how *E. miletus* in higher-altitude adjust to the environment by modifying alterations in gut community and physiological indicators when faced with food restriction in winter. Thus, by employing 16S rRNA gene sequencing technology to examine the adaptation of *E. miletus*’s gut microbiota and physiological markers in various altitude zones under food restriction. It clarified the survival adaption mechanisms of high-altitude *E. miletus* in the face of food scarcity by comparing the variations in gut microbiota with altitude and their interactions with physiology in terms of body mass regulation. We hypothesized that food shortage will affect the composition and diversity of gut microbiota as well as physiological indices of *E. miletus* at high altitude.

## 2 Materials and methods

### 2.1 Collection of experimental animals

*Eothenomys miletus* were collected from Jingdong County (JD) and Xianggelila County (XGLL) in winter of 2023, respectively. Experimental animals were all non-breeding healthy adult individuals. The habitat characteristics of the sampling sites were shown in Table 1.

**Table 1:** Detailed information on the sampling sites of *E. miletus.*

Regions	Sample size	Body mass (mean ± SE)	Geographic location	Altitude/m	Average temperatures (winter)/°C	Precipitation/mm	Vegetation type
JD	21 (♂11, ♀10)	43.7348	99°83′16″ E, 27°90′73″ N	2,217	16.1	597.0	Savannah shrubs
XGLL	19 (♂10, ♀9)	38.3384	100°42′49″ E, 24°90′30″ N	3,321	6.9	984.2	Subalpine meadows

JD, Jingdong County; XGLL, Xianggelila County.

### 2.2 Experimental designs

**Figure 1:**
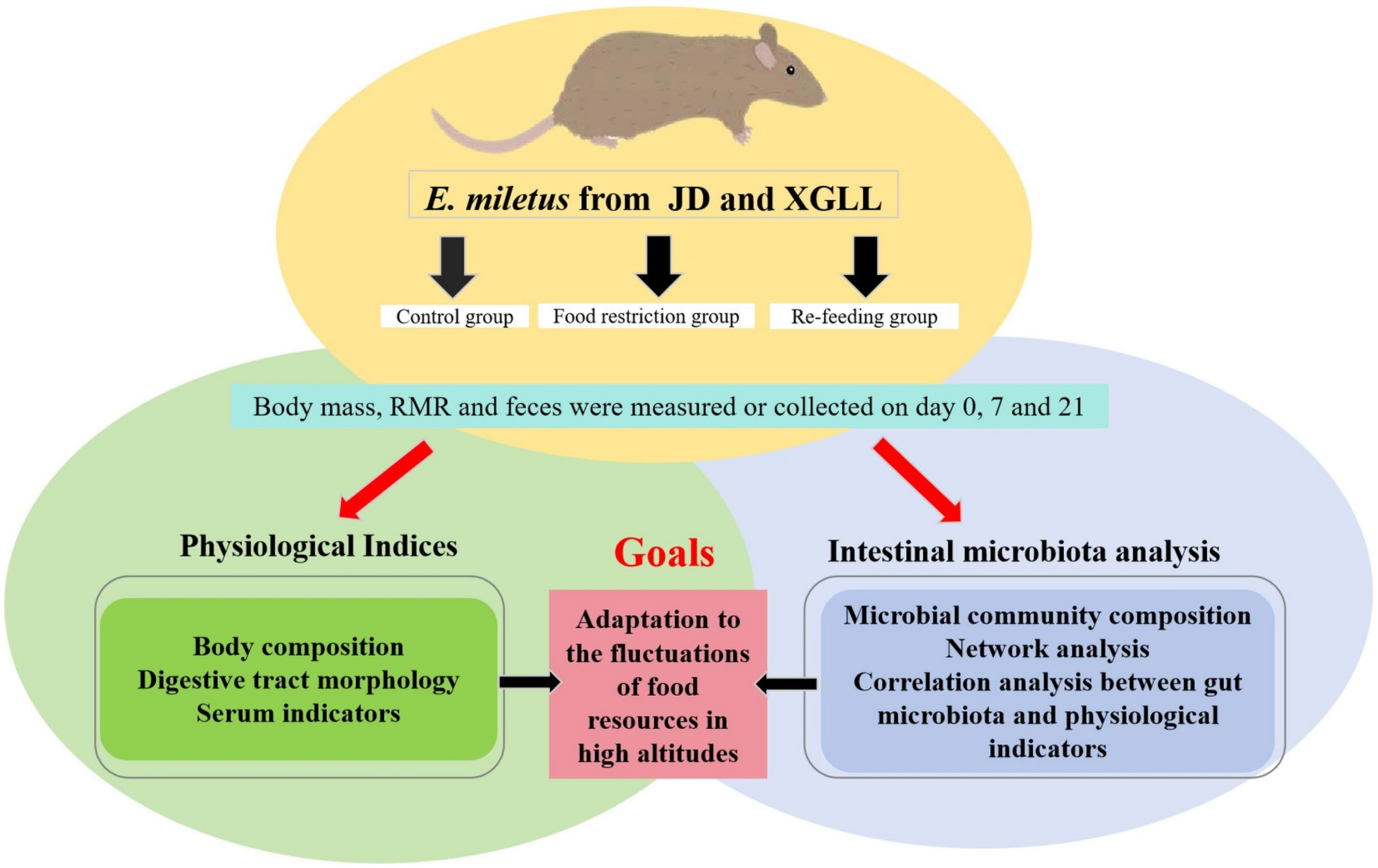
Experimental design.

*Eothenomys miletus* that were captured in two separate locations were sterilized to remove fleas, brought back to the animal breeding room at Yunnan Normal University, and put in separate rat cages (260 × 160 × 150 mm). After 4 days of acclimatization, *E. miletus* were divided into three groups using a two-factor (region × food restriction) experimental design: a 0 d control group, a 7 days food-restricted group, and a 14 days re-feeding group after food restriction (Figure 1). The restricted-food group fed 80% of the control group’s food intake (Yang et al., 2013), while the control group had free access to food and water. That is, JD control group (CJD, *n* = 7), JD restricted food group (FRJD, *n* = 7), JD re-feeding group (FR-ReJD, *n* = 7), XGLL control group (CXGLL, *n* = 7), XGLL restricted food group (FRXGLL, *n* = 7), XGLL re-feeding group (FR-ReXGLL, *n* = 6). Room temperature was controlled at 25 ± 1°C with a photoperiod of 12 L:12D (Light: Dark). The trial period lasted 21 days, during which the animals were fed standard rat chow (from Kunming Medical University, Kunming, China). Body mass, food intake, RMR and feces were measured simultaneously when the test was carried out on day 0, 7, and 21. Body mass was measured with an LT502 electronic balance (accurate to 0.01 g), food intake was measured with the food balance method, and RMR was measured with a portable respirometer (Gong et al., 2022). They were carried out under carbon dioxide anesthesia following the identification of the pertinent markers, and serum and rectal feces were collected.

### 2.3 Measurement of physiological indices

At the end of the experiment, blood was collected, allowed to stand for 1 h in the refrigerator at 4°C, and was centrifuged at 4°C (4,000 r/min, 30 min), taking up serum in a centrifuge tube, stored in the refrigerator (−80°C), and set aside. Measurements of leptin, glucose (Glu), triglyceride (Tg), total cholesterol (Tc), SCFAs, LPS, fasting-induced adipocyte factor (FIAF), and TNF-*α* were measured the serum using an enzyme-linked immunosorbent assay (ELISA). After the experimental animals were executed, brown adipose tissue (BAT) was carefully removed, weighed and put into centrifuge tubes, and stored in a low-temperature refrigerator for storage. The content and activity of uncoupling protein 1 (UCP1) were determined using ELISA. The assay was conducted following the instruction manual, and the product numbers of the assay kits were leptin Assay Kit (JM-11498 M1), Glu Assay Kit(S0104F-1), Tg Assay Kit (S0104F-1) Tg Assay Kit (S0140O-1), Tc Assay Kit (S05042-1), SCFAs Assay Kit (JM-11498 M1), LBP Assay Kit (JM-12488 M1), FIAF Assay Kit (JM-12613 M1), TNF-α AssayKit(JM-02415 M1).

### 2.4 Measurement of digestive tract morphology

Organs and other tissues are separated and then weighted after draining the surface liquid with a filter, (accurate to 0.001 g). After removing the digestive tract, carefully remove the mesentery, connective tissue, and fat from the stomach, small intestine, colon, and cecum. Then, weigh and measure the length of each organ.

### 2.5 DNA extraction and 16S rRNA gene sequencing

A centrifugal column-based soil genome extraction kit (DNeasy®PowerSoil®Kit, Germany) was used to collect the rectal feces and enrich the total DNA on the filter membrane. The concentration of purified PCR products was determined using a Nanodrop 2000 spectrophotometer, and valid samples were defined as having a nucleic acid concentration greater than 10 ng/uL and a purity (A260/A180) greater than 1.8. Purified DNA samples were mixed at equimolar concentrations and sequenced using the Illumina Miseq platform (Illumina, San Diego, CA, USA).

### 2.6 Bioinformatics analysis

The 2 × 250 bp double end sequences were obtained by sequencing on the Illumina Miseq platform (Illumina, San Diego, CA, USA) and these raw data were processed and analyzed using the QIIME platform (version 1.8). The double-end sequences were first spliced using Flash software (version 1.2.111) and then matched to a unique barcode label for each sample. Low quality sequences (sequence length less than 300 or base mass fraction less than 30) were removed during the splicing process. By QIIME software, the raw data was processed, using Flash software to clear low-quality sequences, and then removing the chimeras in the sequences by Usearch 7.0 software. The OTU sequences with more than 97% recognition were clustered using the Uclust algorithm, and the representative OTU sequences were analyzed and identified based on the Ribosomal Database Project, and finally, the sequences of all samples were normalized by the “Daisychopper” script code. Finally, we standardized the sequences of all the samples by using the code “Daisychopper.”

### 2.7 Data analysis

#### 2.7.1 Microbial community composition

A percentage stacked bar chart was created using origin 2018 to describe the bacterial community.

#### 2.7.2 *α* and *β* diversity

α diversity was estimated by 2 diversity indicators: Chao1 and Shannon diversity and described by creating box plots using Origin 2018, and Kruskal-Wallis H-test was used in SPSS 21 to analyze if differences in diversity between the two groups. The reason we chose the non-parametric test here was that these two indicators did not conform to the homogeneity of variance.

β diversity: community structure was described using QIIME and Origin 2018. Based on unweighted and weighted UniFrac distance matrices, PERMANOVA (Permutational multivariate analysis of variance) was used to calculate the difference among the groups. The unweighted UniFrac distance depends on phylogenetic relationships and OTU species abundance, while species absence/presence and phylogenetic relationships are considered by the weighted UniFrac distance. Then principal co-ordinates analysis (PCoA) was used to visualize the β diversity of all samples.

#### 2.7.3 Venn diagram

Common and unique parameters between groups were analyzed via Venn diagrams implemented online in Venn 2.1.

#### 2.7.4 Enrichment analysis

We used one-way of variance analysis to compare the distribution differences of microbial abundance among groups, and the variable was different treatment group. The genera of microorganisms with significant differences in distribution between groups were screened out, and the heatmap was drawn by R packages “vegan,” “permute,” and “gplots” for visualization. The prefixes “o” and “f” represented the order and family level of unidentified genera, respectively.

#### 2.7.5 Heat map of the correlation between environmental physicochemical properties and dominant microorganisms in the feces of *Eothenomys miletus* in different regions

Pearson analysis using SPSS 21 and R3.6.2 were used to obtain the correlation heat map.

#### 2.7.6 RDA analysis

Redundancy analysis (RDA) was used to assess the correlation between dominant genera (top 9) and physicochemical factors using Canoco 5.0.

#### 2.7.7 Network analysis

Use R3.6.2 and Gephiv.0.9.2 software to further analyze these results to generate network analysis (*p* < 0.05, |*r*| > 0.4). R was used to calculate the correlation between these microorganisms, and the network analysis diagram was drawn based on the correlation matrix. With the help of Gephi, we further calculated the topological characteristics (namely modularity) of the network.

#### 2.7.8 Physiological indicators analysis

The data was analyzed using SPSS 26.0 software, and the differences in various indicators between the two regions were analyzed using two-way ANOVA or two-way ANCOVA (Region × Diet), with body mass as the covariate. The results were expressed as mean ± SE, with *p* < 0.05 indicating significant differences.

## 3 Results

In the present study, a total of 39 samples were collected to extract the DNA and amplify the PCR products, and each sample was normalized to 6,982 sequences after removing low-quality sequences, chimeras, monomers, and chloroplasts.

### 3.1 Effects of food restriction on physiological indicators

**Figure 2:**
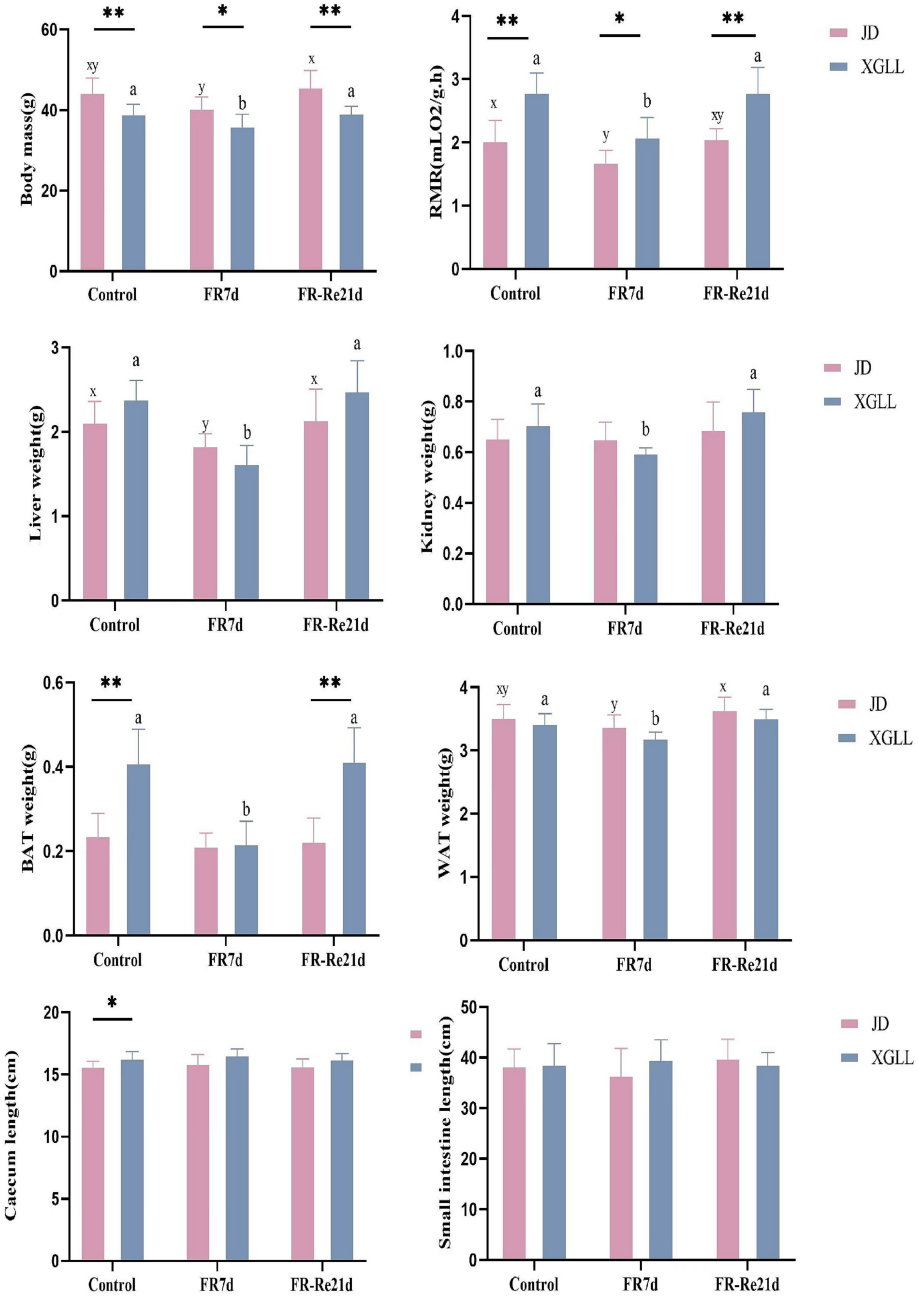
Effects of different regions and food restriction on physiological indices of *E. miletus*. Control, Control group; FR7d, Restricted food for 7 days group; FR-Re21d, 14 days re-feeding group after food restriction.

**Figure 3:**
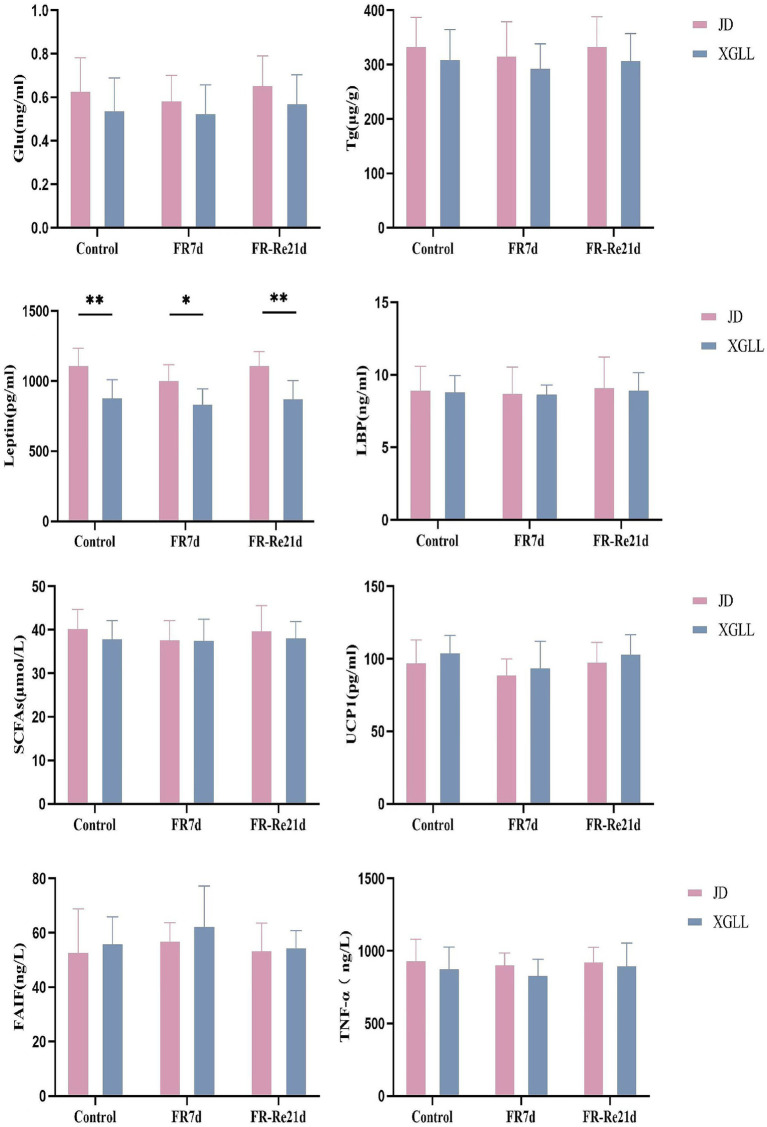
Effect of different regions and food restriction on serum indices of *E. miletus*. Control, Control group; FR7d, Restricted food for 7 days group; FR-Re21d, 14 days re-feeding group after food restriction. Data were mean ± standard deviation. Different letters indicated significant differences between treatments in the same region, xy indicates JD region and ab indicates XGLL region. *p < 0.05, **p < 0.01, JD and XGLL were compared on the same day.

Body mass of *E. miletus* was significantly affected by area and time (Region: *F* = 35.751, *p* < 0.01; Time: *F* = 7.895, *p* < 0.05). Region and time significantly affected RMR of *E. miletus* (Region: *F* = 15.928, *p* < 0.01; Time: *F* = 17.141, *p* < 0.01). The interaction of region and time significantly affected liver weight in *E. miletus* (Region: *F* = 32.960, *p* < 0.01; Time: *F* = 8.225, *p* < 0.01; Interaction: *F* = 12.614, *p* < 0.01). Region significantly affected kidney weight and lung weight in the *E. miletus* (Region: *F* = 14.679, *p* < 0.05; Time: *F* = 12.245, *p < 0.05*). Cecum length was significantly affected by region and time (Region: *F* = 21.508, *p* < 0.01; Time: *F* = 3.321, *p* < 0.05). Time significantly affected WAT weight (*F* = 4.744, *p* < 0.05) and the interaction of region and time significantly affected BAT weight (Region: *F* = 32.618, *p* < 0.01; Time: *F* = 7.154, *p* < 0.05; and Interaction: *F* = 10.955, *p* < 0.01) in *E. miletus* (Figure 2). Region significantly affected the Leptin content of the *E. miletus* (*F* = 18.688, *p* < 0.01) (Figure 3). Region and time did not significantly affect other indices of *E. miletus* in XGLL (Table 2). *E. miletus* in XGLL had smaller changes in leptin, larger changes in RMR, greater weight loss in main organs, and larger changes in cecum.

**Table 2:** Effects of area and food restriction on other physiological indices of *E. miletus.*

Parameter	JD			XGLL			Region		Time		Region × Time	
	Con group *n* = 7	FR group *n* = 7	FR-Re group *n* = 7	Con group *n* = 7	FR group *n* = 7	FR-Re group *n* = 7	*F*	*P*	*F*	*P*	*F*	*P*
Food intake (g/d)	8.203 ± 1.276	6.562 ± 1.021	8.050 ± 0.595	11.21 ± 0.992	9.109 ± 0.767	11.605 ± 1.576	46.895	**<0.01**	7.477	**<0.05**	0.873	0.427
Heart weight (g)	0.282 ± 0.021	0.236 ± 0.008	0.284 ± 0.052	0.314 ± 0.052	0.246 ± 0.021	0.333 ± 0.044	23.840	**<0.01**	3.661	**<0.05**	2.382	0.108
Spleen weight (g)	0.110 ± 0.118	0.091 ± 0.016	0.092 ± 0.025	0.130 ± 0.035	0.065 ± 0.013	0.109 ± 0.027	6.248	**<0.05**	5.960	**<0.05**	5.757	**<0.05**
Lung weight (g)	0.323 ± 0.061	0.306 ± 0.031	0.323 ± 0.057	0.342 ± 0.034	0.299 ± 0.038	0.395 ± 0.067	12.245	**<0.05**	0.942	0.400	3.674	**<0.05**
Stomach length (cm)	2.286 ± 0.403	2.137 ± 0.420	2.017 ± 0.321	2.057 ± 0.321	2.033 ± 0.186	2.083 ± 0.248	0.108	0.744	0.466	0.632	0.683	0.512
T c (ug/g)	106.018 ± 20.959	103.975 ± 18.977	111.505 ± 15.242	107.123 ± 3.386	102.720 ± 21.635	107.594 ± 23.922	0.337	0.565	0.051	0.950	0.042	0.959

*P < 0.05 indicates significant difference, P < 0.01 indicates extremely significant difference.*

Data were mean ± standard deviation. Different letters indicated significant differences between treatments in the same region, xy indicates JD region and ab indicates XGLL region. *p < 0.05, **p < 0.01, JD and XGLL were compared on the same day.

### 3.2 Microbial community composition

**Figure 4:**
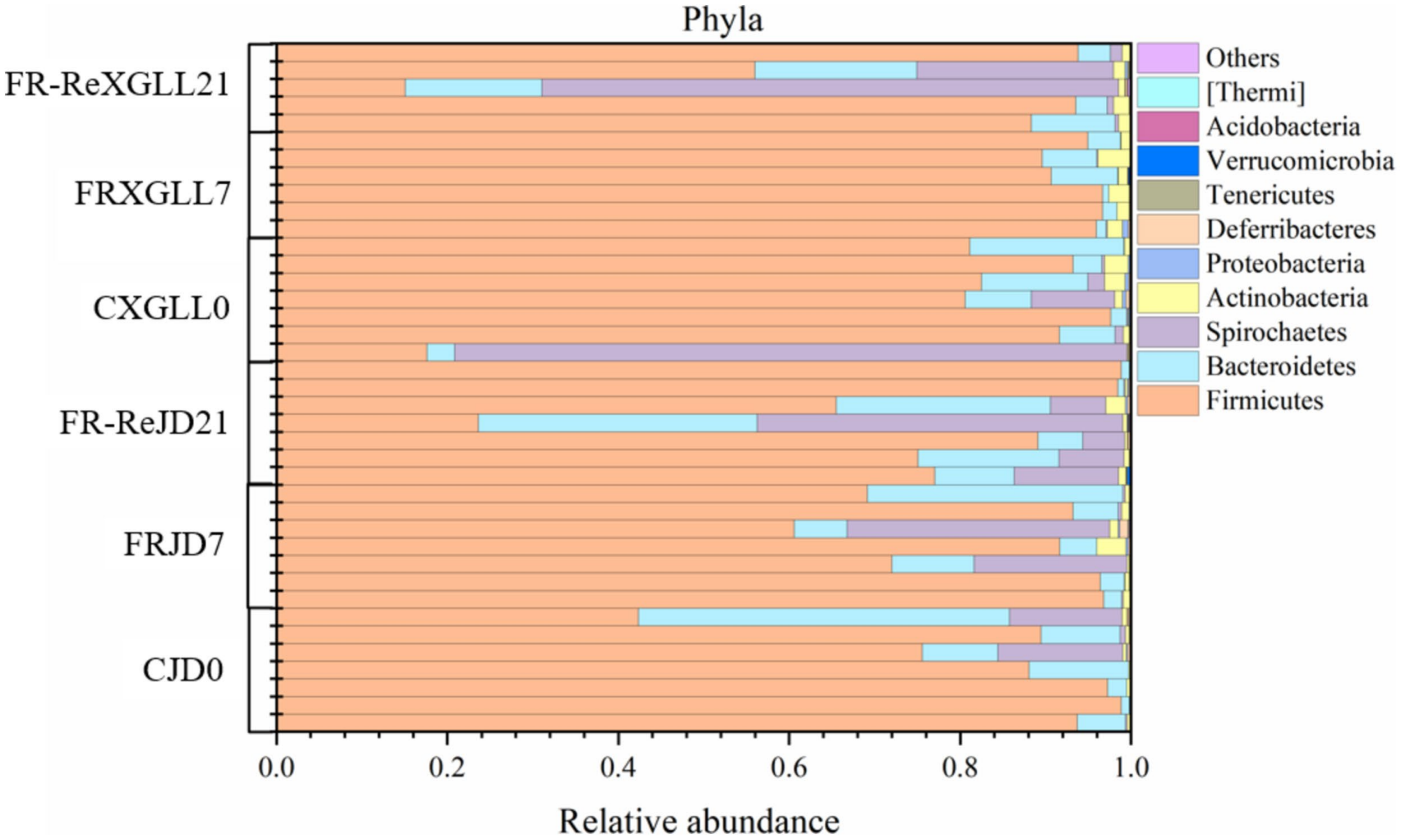
Microbial phylum level community composition. CJD0, JD control group; FRJD7, JD restricted food group; FR-ReJD21, JD re-feeding group; CXGLL, XGLL control group; FRXGLL7, XGLL restricted food group; FR-ReXGLL21, XGLL re-feeding group.

Firmicutes, Bacteroidetes, and Spirochaetes were the fecal microbial dominant phylum of *E. miletus* (Figure 4), with mean relative abundances of 80.71, 9.22, and 8.66%, respectively. The relative abundance of Firmicutes, the predominant phylum of fecal microbes in *E. miletus*, did not differ significantly between the two regions of JD and XGLL, similar to the relative abundance of Bacteroidetes did not change between the regions at different treatment periods. Conversely, the dominant phylum Spirochaetes’ average relative abundance in XGLL exhibited a tendency of decline followed by an increase, while the shift was less noticeable in JD.

**Figure 5:**
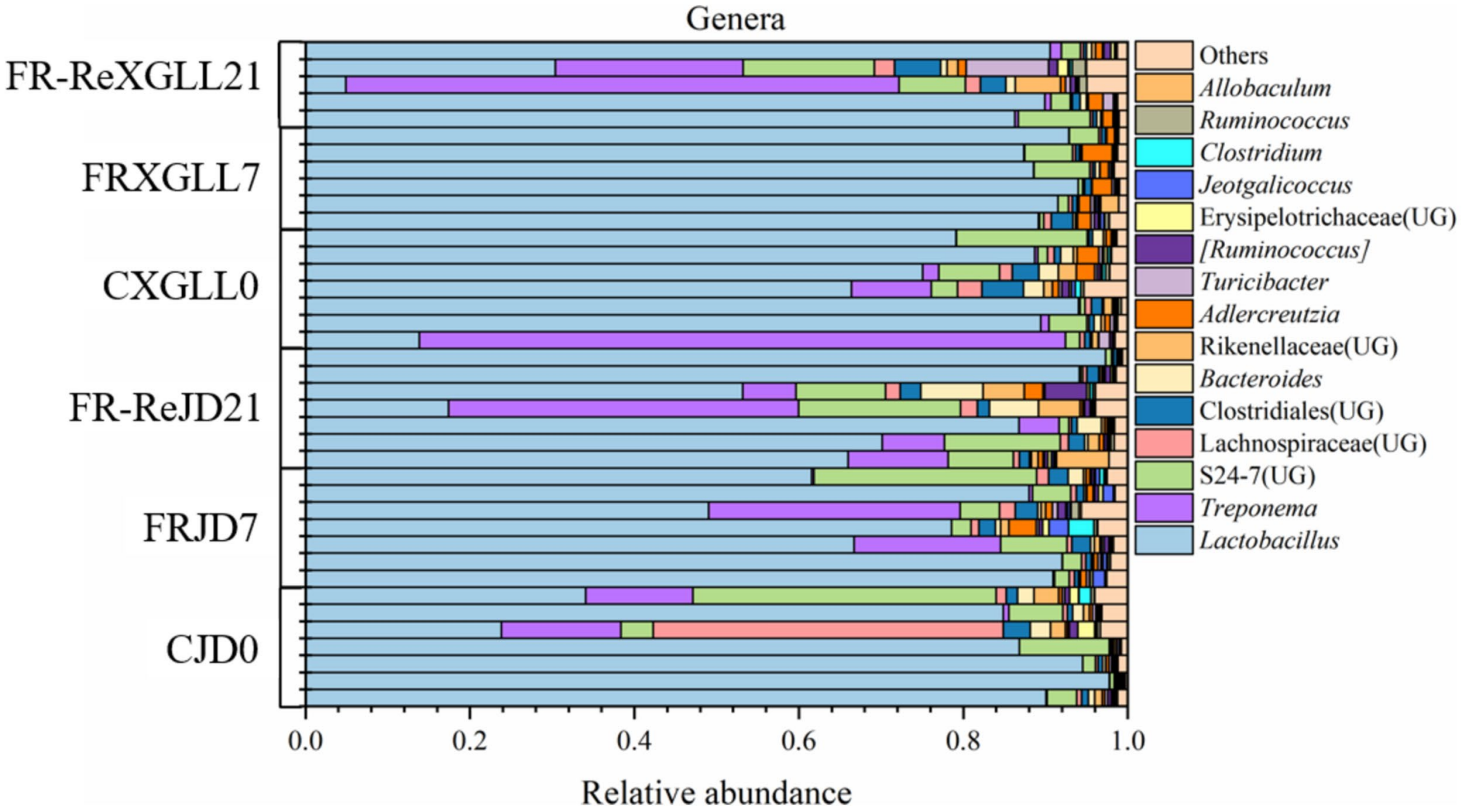
Microbial composition at the genus level. CJD0, JD control group; FRJD7, JD restricted food group; FR-ReJD21, JD re-feeding group; CXGLL, XGLL control group; FRXGLL7, XGLL restricted food group; FR-ReXGLL21, XGLL re-feeding group.

At the genus level, the main dominant genera of fecal microorganisms in the *E. miletus* were *Lactobacillus*, *Treponema*, and S24-7 (UG), with average relative abundances of 73.72, 8.63, and 6.68% in all groups (Figure 5). *Lactobacillus* did not exhibit a significant variation in mean relative abundance over time or place in *E. miletus*. In XGLL, the dominating genus *Treponema*’s average relative abundance first trended downward before rising.

### 3.3 Microbial community *α* and *β* diversity analysis

**Figure 6:**
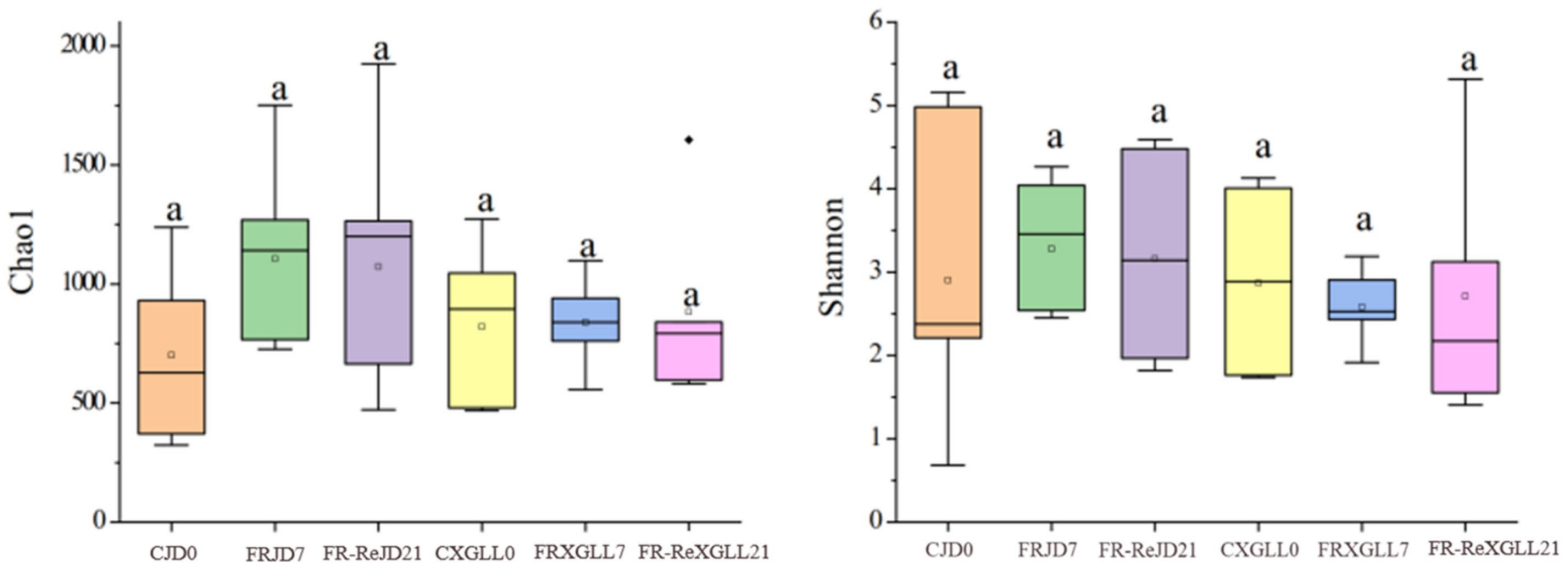
Microbial *α*-diversity of fecal microorganisms of *E. miletus* in different regions and time periods. CJD0, JD control group; FRJD7, JD restricted food group; FR-ReJD21, JD re-feeding group; CXGLL, XGLL control group; FRXGLL7, XGLL restricted food group; FR-ReXGLL21, XGLL re-feeding group.

**Figure 7:**
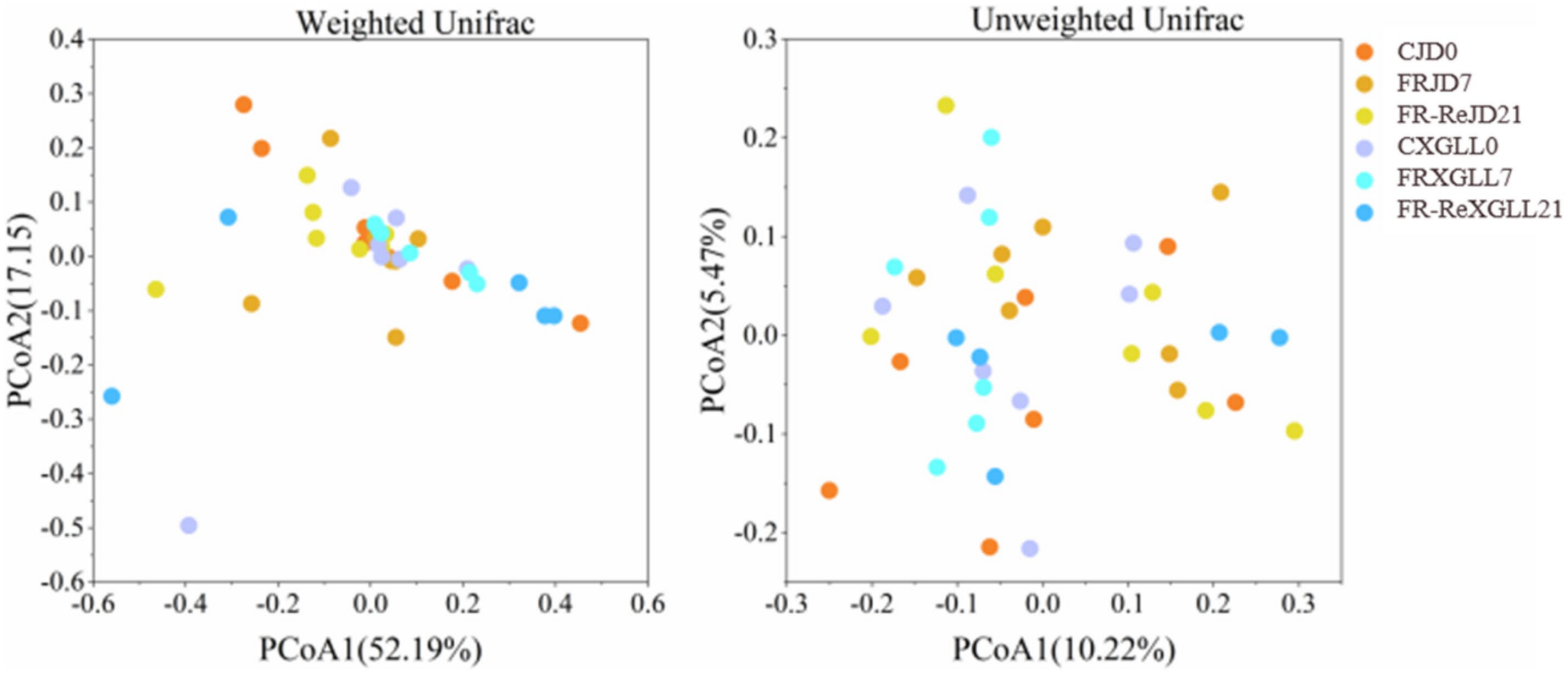
*β*-diversity of fecal microorganisms of *E. miletus* in different regions and periods in the food-restricted group. CJD0, JD control group; FRJD7, JD restricted food group; FR-ReJD21, JD re-feeding group; CXGLL, XGLL control group; FRXGLL7, XGLL restricted food group; FR-ReXGLL21, XGLL re-feeding group.

Food restriction did not significantly affect the α-diversity (Chao1 and Shannon diversity) of *E. miletus* (Figure 6). The PCoA plot showed that the β-diversity of *E. miletus* in the various food-restricted group regions was dispersed, without any discernible tendency of aggregation (Figure. 7), which implied that the fecal microbial β-diversity of *E. miletus* was not significantly affected by the food-restricted treatment.

Subsequent PERMANOVA testing revealed that the overall distribution of fecal microbial β-diversity (weighted and unweighted matrices) in *E. miletus* was not significantly affected by treatment or location (Table 3).

**Table 3:** PERMANOVA test for fecal microorganisms in different regions and periods of time of *E. miletus.*

PERMANOVA	Weighted Unifrac			Unweighted Unifrac		
	*F*	*R^2^*	*P*	*F*	*R^2^*	*P*
CJD0 vs. FRJD7	2.024	0.144	0.149	0.893	0.069	0.469
FRJD7 vs. FR-ReJD21	1.031	0.079	0.391	0.690	0.054	0.706
CXGLL0 vs. FRXGLL7	0.421	0.037	0.731	0.993	0.083	0.401
FRXGLL7 vs. FR-ReXGLL21	0.063	0.007	0.994	0.719	0.074	0.712
CJD0 vs. CXGLL0	1.844	0.133	0.156	0.485	0.039	0.927
FRJD7 vs. FRXGLL7	0.244	0.022	0.853	0.565	0.049	0.877
FR-ReJD21 vs. FR-ReXGLL21	0.291	0.028	0.837	0.991	0.090	0.392

*P < 0.05 indicates significant difference; CJD0, JD control group; FRJD7, JD restricted food group; FR-ReJD21, JD re-feeding group; CXGLL, XGLL control group; FRXGLL7, XGLL restricted food group; FR-ReXGLL21, XGLL re-feeding group.*

### 3.4 Distribution of common and unique microorganisms in different regions and periods

**Figure 8:**
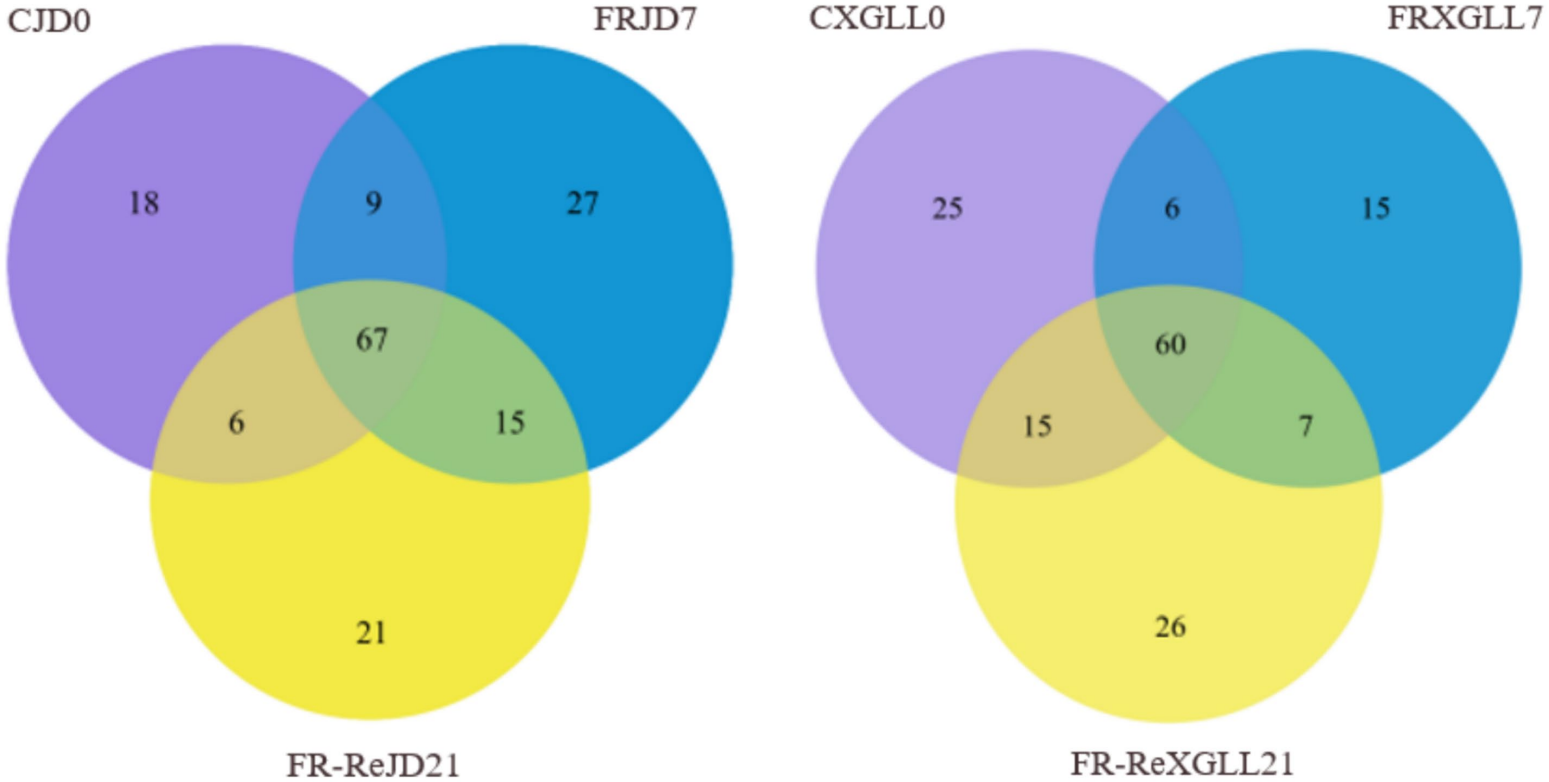
Venn diagram of fecal microorganisms of *E. miletus* feces at different time periods. CJD0, Jingdong control group; FRJD7, Jingdong restricted food group; FR-ReJD21, Jingdong re-feeding group; CXGLL, Xianggelia control group; FRXGLL7, Xianggelila restricted food group; FR-ReXGLL21, Xianggelila re-feeding group.

There were 67 genera of fecal microorganisms in the food-restricted group of *E. miletus* in the JD (Figure 8). Among them, 18 genera were unique to CJD0 group, 27 genera were unique to the FRJD7 group, and 21 genera were unique to FR-ReJD21 group. The food-restricted group of *E. miletus* at XGLL had 60 genera of fecal bacteria. In XGLL, there were 31 genera that were particular to the CXGLL0 group, 15 genera that were specific to FRXGLL7 group, and 26 genera that were exclusive to FR-ReXGLL21 group. It showed that the common and endemic bacteria in XGLL and JD were almost identical.

**Figure 9:**
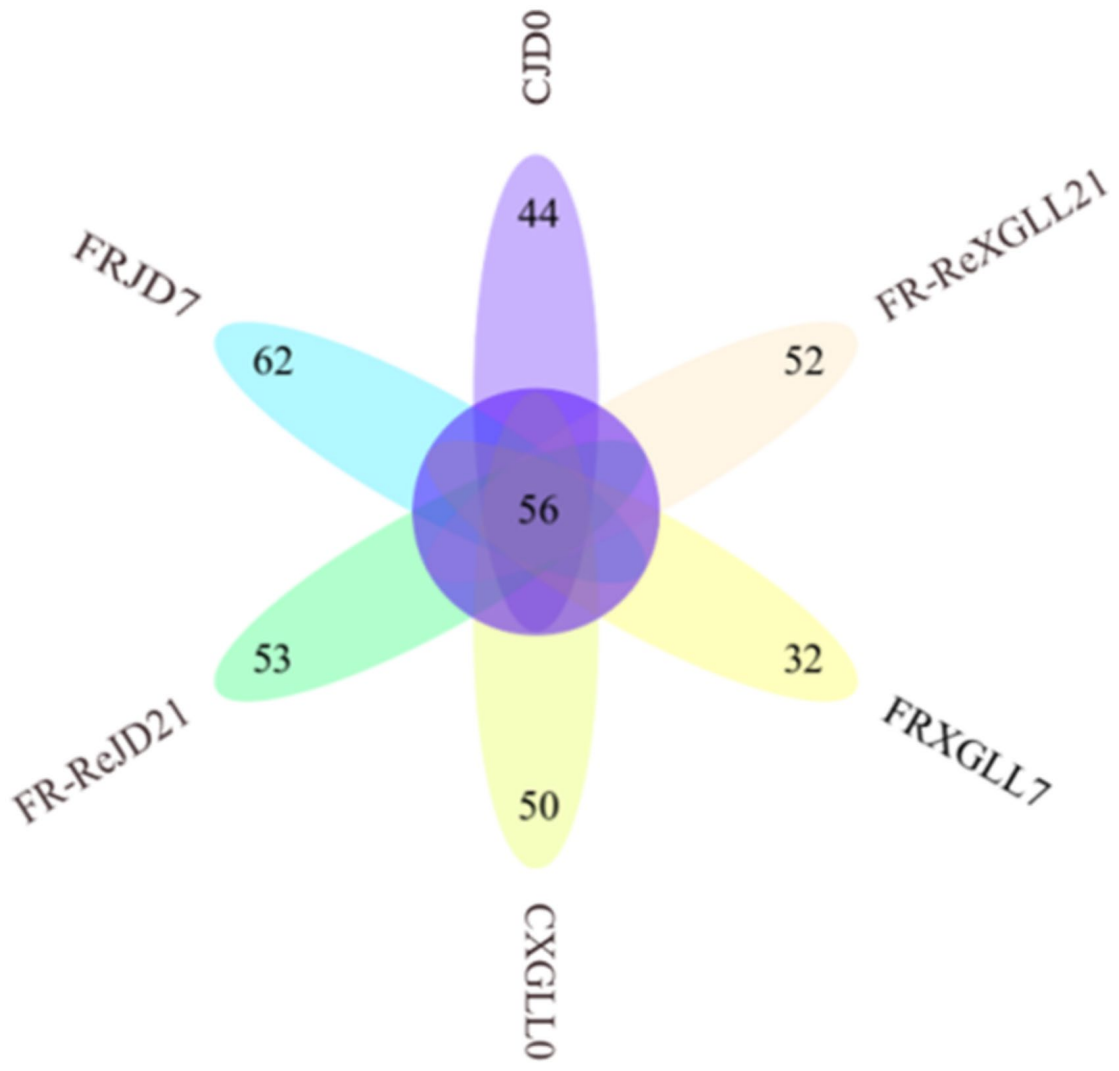
Venn diagram of fecal microorganisms of *E. miletus* in different regions and time periods. CJD0, JD control group; FRJD7, JD restricted food group; FR-ReJD21, JD re-feeding group; CXGLL, XGLL control group; FRXGLL7, XGLL restricted food group; FR-ReXGLL21, XGLL re-feeding group.

There were 56 genera of fecal bacteria in various places and periods as Figure 9 demonstrated. Among them, 44 genera were exclusive to CJD0 group, 62 genera were exclusive to FRJD7 group, and 53 genera were exclusive to the FR-ReJD21 group. In XGLL, there were 50 genera were exclusive to CXGLL0 group, 32 genera were exclusive to FRXGLL7 group, and 52 genera were exclusive to FR-ReXGLL21 group.

### 3.5 Analysis of microbial enrichment differences in different regions and periods

**Figure 10:**
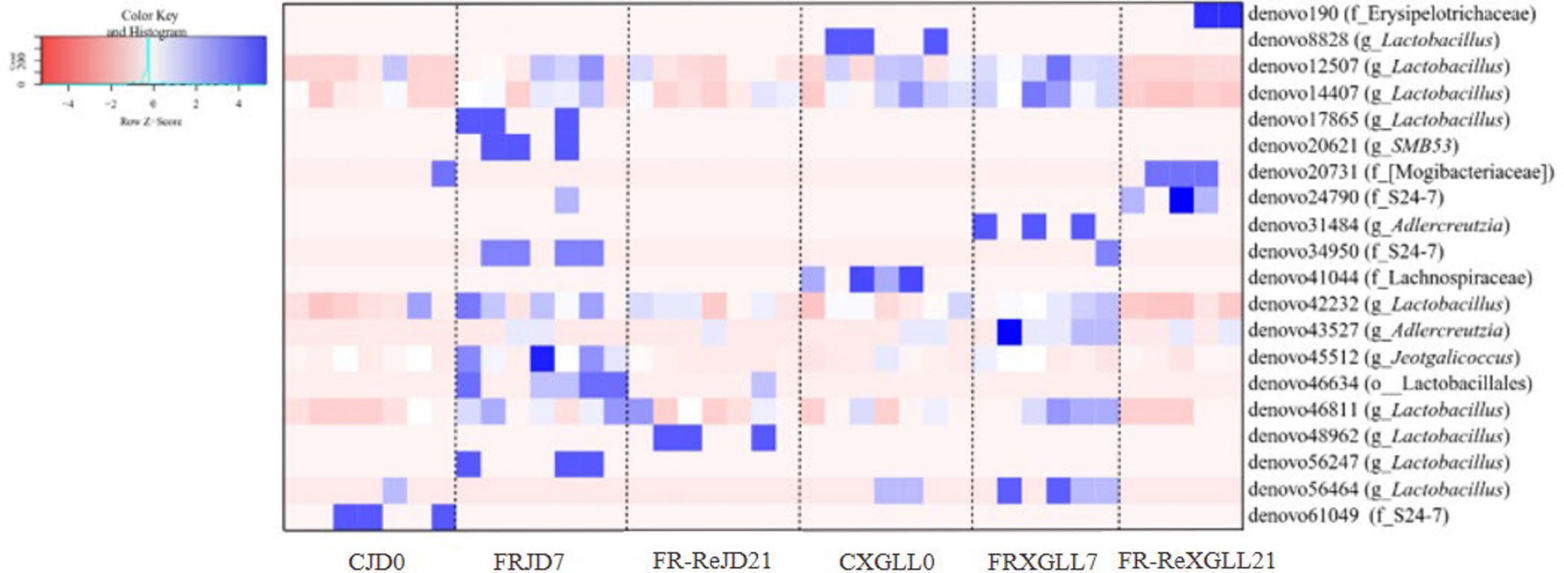
Differential microbial analysis of the feces of *E. miletus* in the food-restricted groups in Jindong and Xianggelila at different periods of time. CJD0, JD control group; FRJD7, JD restricted food group; FR-ReJD21, JD re-feeding group; CXGLL, XGLL control group; FRXGLL7, XGLL restricted food group; FR-ReXGLL21, XGLL re-feeding group.

The relative abundance of microorganisms differed in the feces of JD and XGLL *E. miletus* as shown in Figure 10. Denovo61049 (f_S24-7) was most enriched in JD control group (*p < 0.05*). The majority of microbial OTUs, including denovo17865 (g_*Lactobacillus*), denovo20621 (g_SMB53), denovo34950 (f_S24-7), denovo45512 (g_*Jeotgalicoccus*), etc., were higher enriched in the FRJD7 group (*p < 0.05*). Compared to the CJD0 and FRJD7 groups, denovo56247 (g_*Lactobacillus*) was significantly enriched in FR-ReJD21 groups (*p < 0.05*).

In XGLL, OTUs enriched in the CXGLL0 group included denovo8828 (g_*Lactobacillus*) and denovo41044 (f_Lachnospiraceae). Comparing the CXGLL0 and FRXGLL7 groups, denovo190 (f_Erysipelotrichaceae), denovo20731 (f_[Mogibacteriaceae]), and denovo24790 (f_S24-7) were significantly enriched (*p < 0.05*) in FR-ReXGLL21 groups. Similarly to JD, microbial OTUs were more enriched in the 7-day group, such as denovo31484 (g_*AJDercreutzia*), denovo34950 (f_S24-7), denovo43527 (g_*AJDercreutzia*), and denovo46811 (g_ *Lactobacillus*), etc.

### 3.6 Relationship between physiological indicators and microorganisms in different regions and periods in *Eothenomys miletus*

**Figure 11:**
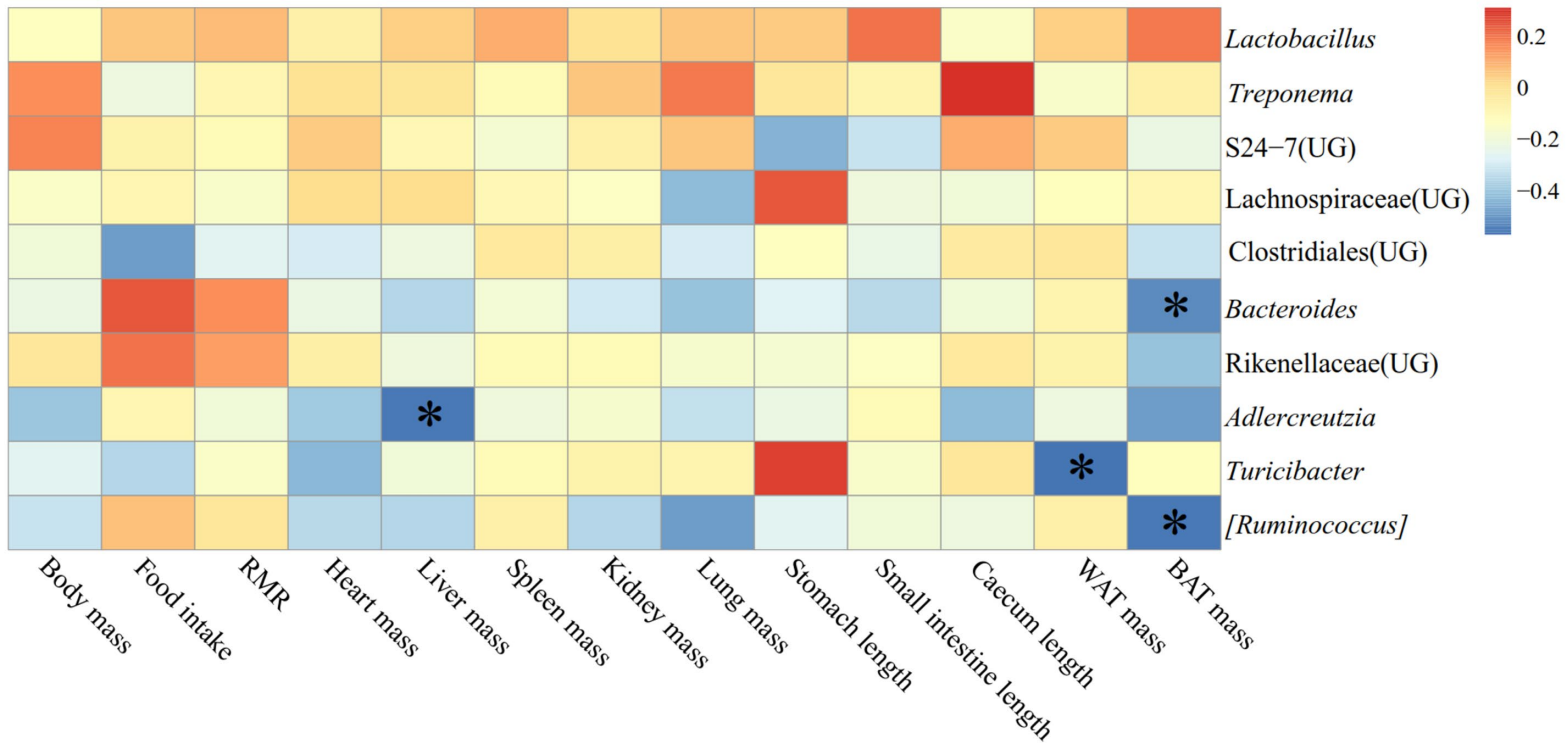
Heat map of physiological indicators related to the dominant microorganisms in the feces of *E. miletus* in JD.

The correlation between physiological indicators and the dominant genera (top ten relative abundance of all samples) in the feces of *E. miletus* in JD was shown in Figure 11. It was found between various physiological indicators and some of the main genera found in the feces of *E. miletus* in JD. For instance, there was a significant negative relationship between liver weight and *AJDercreutzia* abundance (*p* < 0.05), a significant negative correlation between BAT mass and *Bacteroides* and *[Ruminococcus]* abundance (*p* < 0.05), and a significant negative correlation between WAT mass and *Turicibacter* abundance (*p* < 0.05).

**Figure 12:**
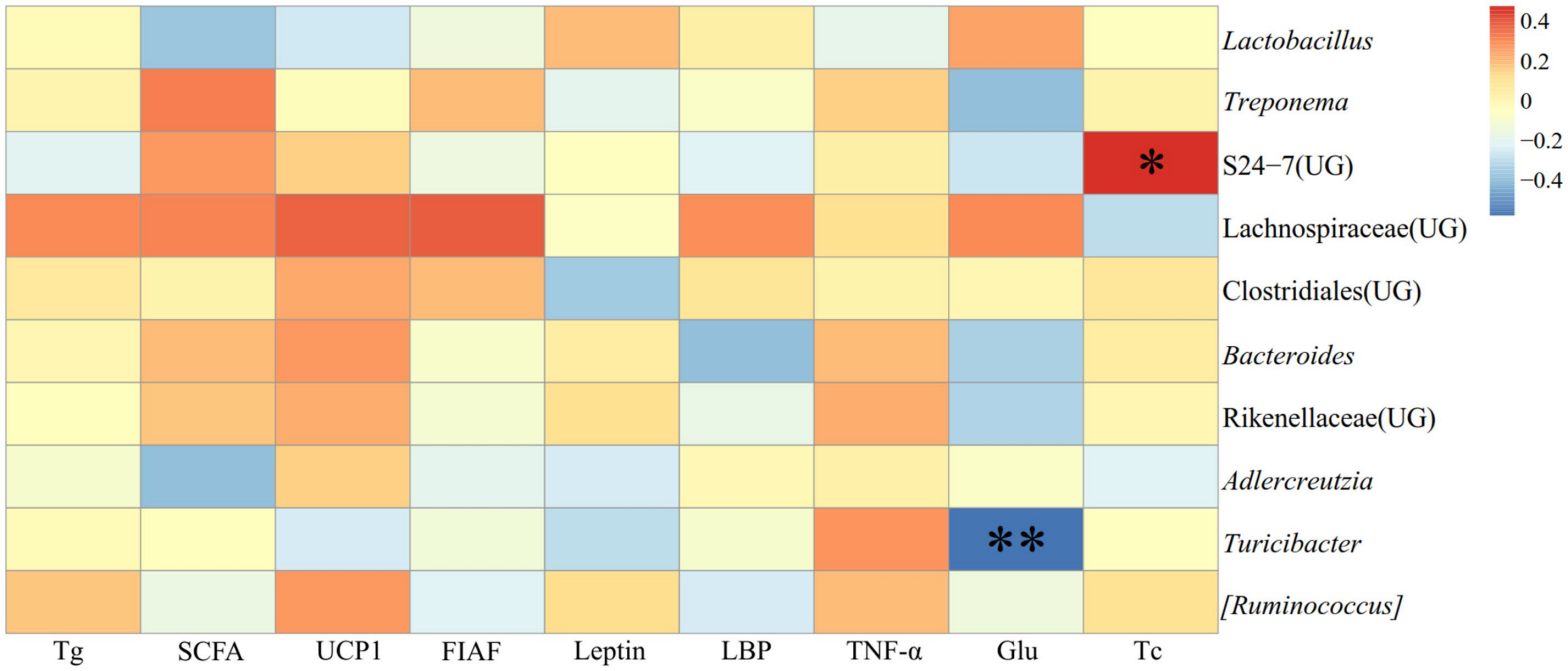
Heat map of the correlation between the detection indicators and the dominant microorganisms in the feces of *E. miletus* in JD.

In JD, it illustrated the relationship between detection markers and fecal dominating genera (top ten relative abundance of all samples) of *E. miletus* (Figure 12). There was a substantial and positive correlation (*p* < 0.05) between Tc and the abundance of S24-7 (UG); conversely, there was a significant and negative correlation (*p* < 0.01) between Glu and *Turicibacter*.

**Figure 13:**
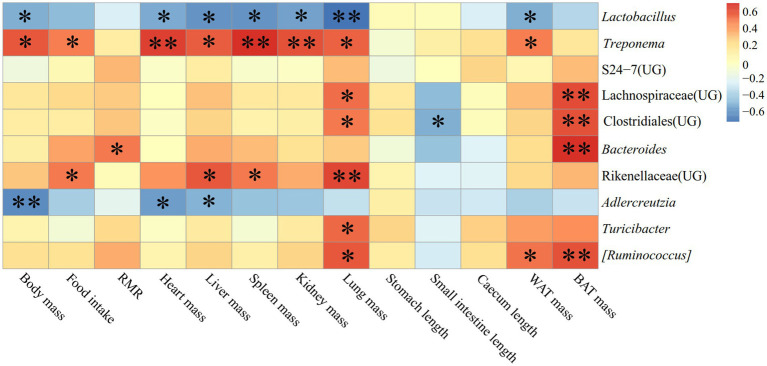
Heat map of physiological indicators related to the dominant microorganisms in the feces of *E. miletus* in XGLL.

Figure 13 displayed the relationship between physiological markers and fecal dominating genera (the top ten relative abundances of all samples) for *E. miletus* in XGLL. In comparison to the JD, XGLL had more physiological indications linked to the fecal dominating genus of *E. miletus.* Among them, *Lactobacillus* abundance was significantly negatively correlated (*p < 0.05*) with the majority of the physiological indicators (body mass, heart weight, liver weight, spleen weight, kidney weight, lung weight, WAT mass), whereas in contrast, *Treponema* abundance was significantly positively correlated (*p* < 0.05) with most of the physiological indicators (body mass, food intake, heart weight, liver weight, spleen weight, kidney weight, lung weight, WAT mass); BAT mass was significantly positively correlated (*p* < 0.05) with the abundance of Lachnospiraceae (UG), Clostridiales (UG), *Bacteroides*, and *[Ruminococcus]*. The abundance of *AJDercreutzia* was significantly negatively correlated (*p* < 0.05) with body mass, heart weight, and liver weight, and the abundance of Rikenellaceae (UG) was positively correlated (*p* < 0.05) with the abundance of food intake, liver weight, spleen weight, and lung weight. Furthermore, it showed a substantial positive correlation (*p* < 0.05) between lung weight and the majority of prominent species, including *Treponema*, Lachnospiraceae (UG), Clostridiales (UG), Rikenellaceae (UG), *Turicibacter*, and [*Ruminococcus*].

**Figure 14:**
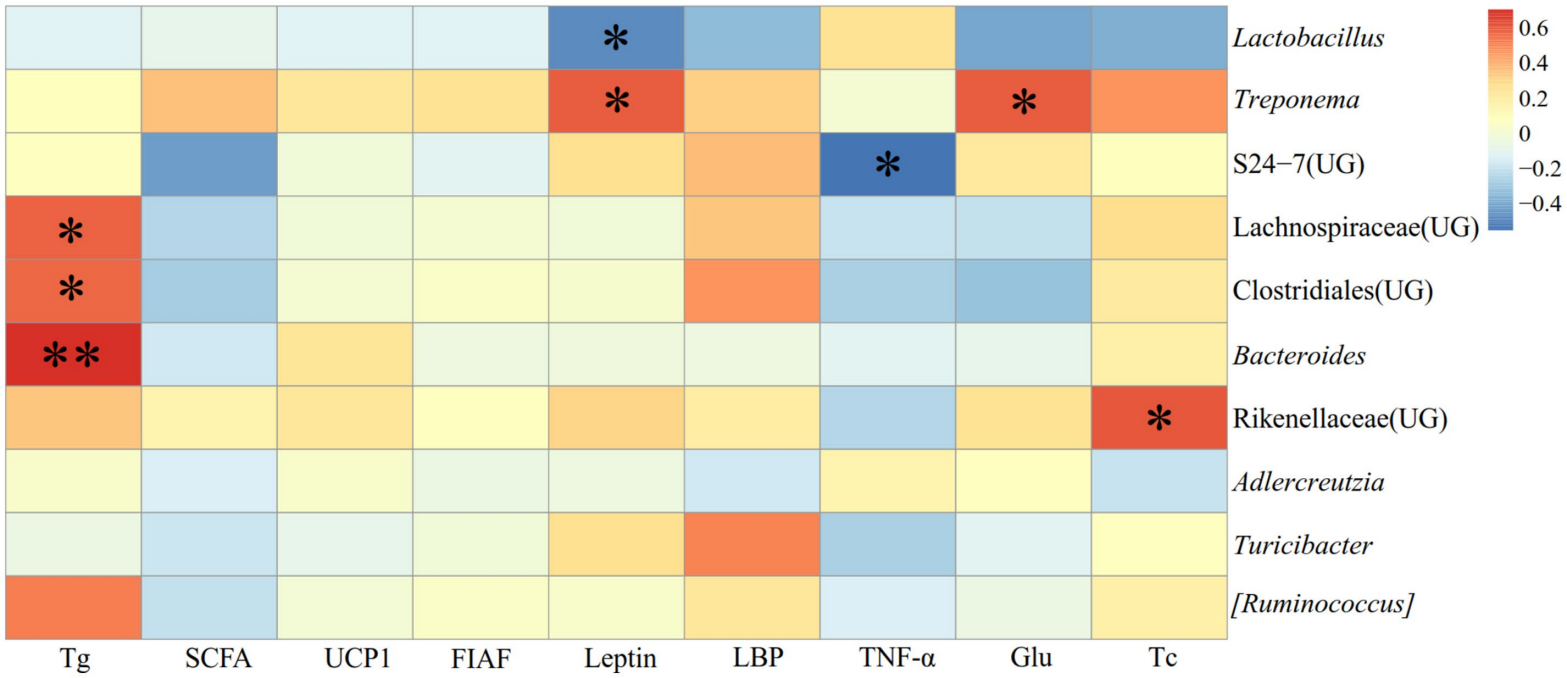
Heat map of the correlation between detected indicators and the dominant microorganisms in the feces of *E. miletus* in XGLL.

Figure 14 illustrated the relationship between the detection indexes and the dominating genera (top ten relative abundance of all samples) in *E. miletus* in XGLL. TNF-*α* was significantly negatively correlated (*p* < 0.05) with S24-7 (UG) abundance; Glu and Tc were significantly positively correlated (*p* < 0.05) with *Treponema* and Rikenellaceae (UG) abundance; Tg was significantly and positively correlated (*p* < 0.05) with the abundance of Lachnospiraceae (UG), Clostridiales (UG), and *Bacteroides*; Leptin was significantly and negatively correlated (*p* < 0.05) with the abundance of *Lactobacillus* and significantly and positively correlated with the abundance of *Treponema*.

**Figure 15:**
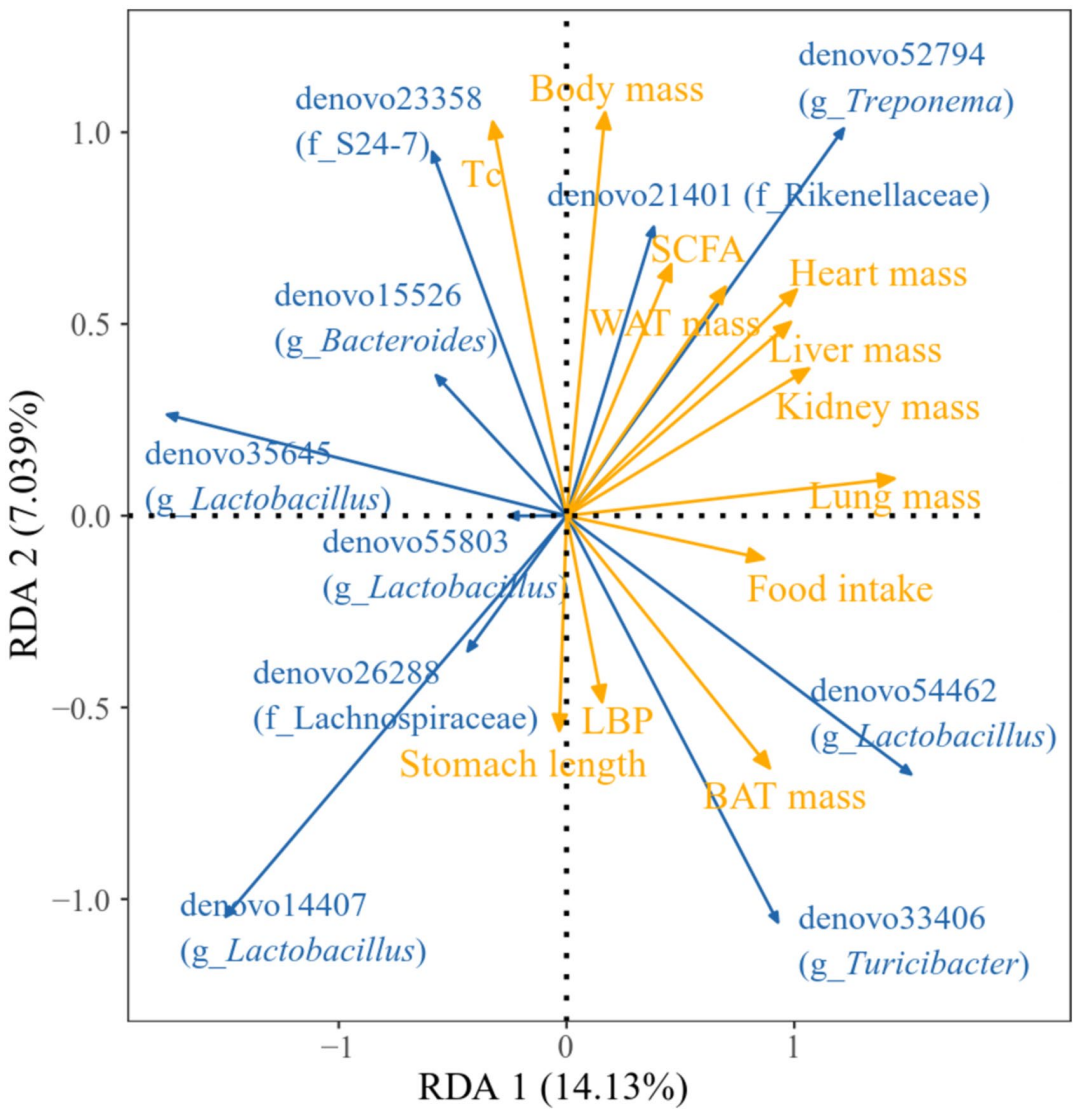
Redundancy analysis (RDA) of correlations between physiological indicators and dominant microbial communities. Blue arrows represent gut microbiota genera, yellow arrows represent physiological markers. The length of the physiological markers arrow can represent the influence of the factor on the gut microbiota. The angles between the arrows represent positive and negative correlations.

Figure 15 illustrated the relationship between various regional physiological markers and the fecal dominance OTUs in *E. miletus*. For instance, the abundance of denovo35645 (g_*Lactobacillus*) was negatively linked with food intake, lung weight, and BAT mass. The denovo52794 (g_*Treponema*) abundance showed a positive correlation with heart weight, liver weight, kidney weight, and lung weight, while the denovo14407 (g_*Lactobacillus*) abundance showed a negative correlation. Lung weight and BAT mass were positively correlated with denovo33406 (g_*Turicibacter*) abundance.

### 3.7 The fecal microbial co-occurrence network of *Eothenomys miletus*

**Figure 16:**
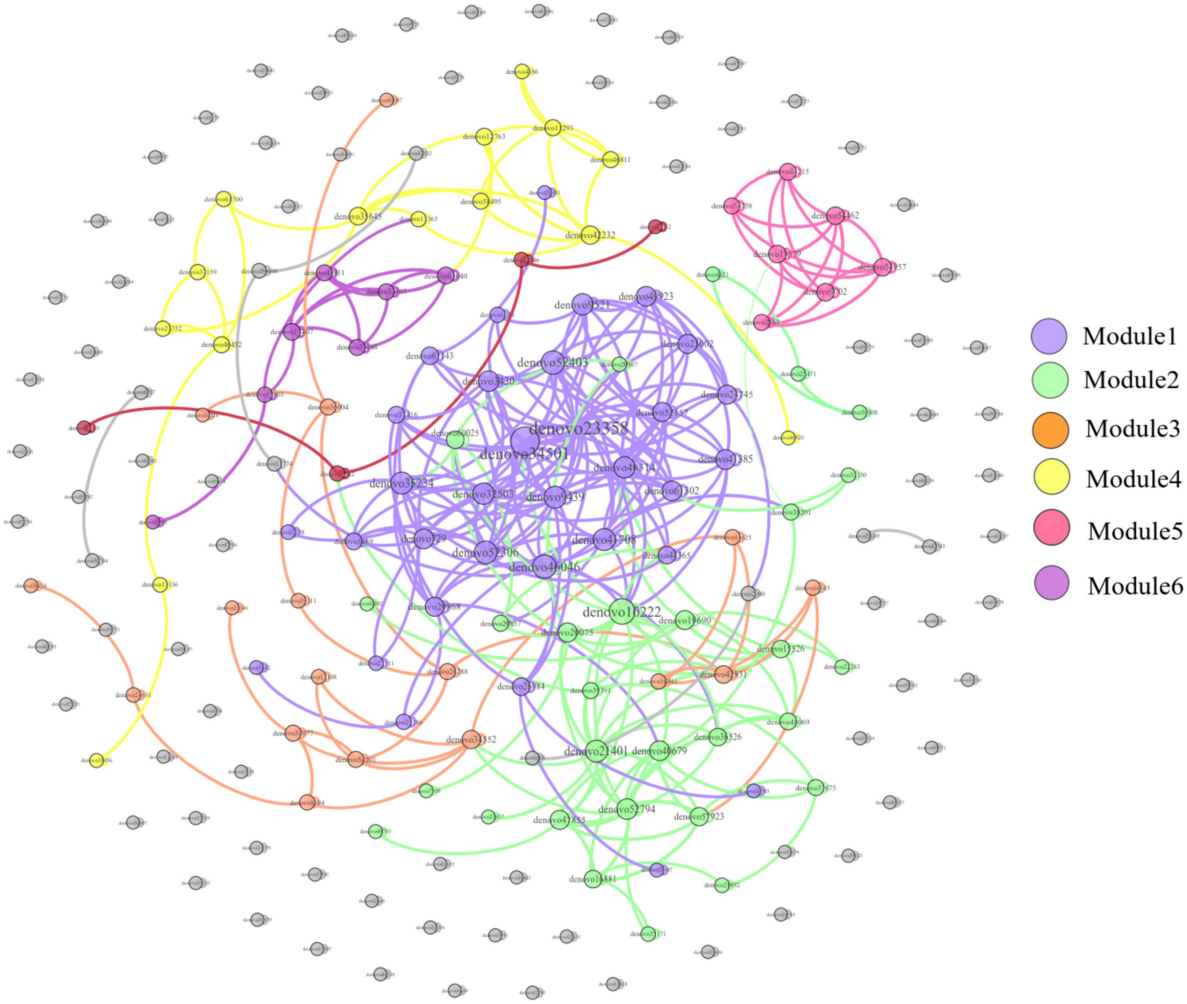
Dominant OTU co-occurrence network of feces of *E. miletus* in all regions. The size of each node is proportional to the number of connections, each module is presented as a specific color.

The correlation network of the dominant OTUs found in the feces of *E. miletus* from various regions as shown in Figure 16. In this network, the top 200 OTUs by relative abundance were included. Gephi 0.9.2 was used to create a network with 200 nodes and 502 edges, of which 501 were positive and 1 was negative, suggesting that the microorganisms in the co-occurring network of this dominant OTU primarily interact cooperatively.

## 4 Discussion

### 4.1 *Eothenomys miletus* in high-altitude had smaller body weights, higher RMR, longer cecums, greater BAT weights, and lower leptin levels under restricted food conditions

*Eothenomys miletus* in JD and XGLL had drastically different body mass, RMR, cecum length, BAT weight, and leptin level in the control group. XGLL had a higher elevation compared to JD, and smaller body mass of *E. miletus* may suggest that it has some degree of size adaption in high-altitude regions. Since body size affects practically all physiological, behavioral, and ecological feature of mammals, changes in body size are typically a crucial approach for adapting to rapidly changing environmental situations (Searing et al., 2023). Rats’ larger lung-to-body-weight ratio might help maintaining respiratory efficiency in an oxygen-sparse high-altitude environment by improving the body’s capacity to absorb, transport, and diffuse oxygen (Shao et al., 2010). Furthermore, higher RMR indicated that rats use more energy to sustain body temperature, perform vital functions, and cope with food scarcity in the high-altitude setting. For small and winter-active rats to survive in high-elevation environments, sustained aerobic thermogenesis is necessary (Storz et al., 2019). The cecum contains fiber hydrolase (Frias et al., 2023), and a longer cecum may indicate that *E. miletus* consume more cellulose at high elevations, which calls for longer cecums to facilitate the digestion of cellulose. The larger BAT weight suggested that *E. miletus* need more calories to stay warm while at high altitudes. Lower levels of the hormone leptin, which controls hunger and energy metabolism and is mostly produced by adipocytes (Perakakis et al., 2021), and lower levels of leptin may indicate *E. miletus* had an increased appetite at high altitude and need to consume more food to satisfy their energy requirements. After re-feeding, larger changes in body mass, RMR and liver weight were observed in JD than in XGLL, which may be related to their larger body mass. We suggesting that different physiological adaptation may be used by *E. miletus* living in the XGLL to cope with food restriction. These strategies could include improving energy use pathways and lowering activity levels to avoid needless energy consumption.

### 4.2 Reduced *α* diversity of gut microorganisms and number of OTUs in high altitude *Eothenomys miletus* under restricted food conditions

In this experiment, the dominant phyla in *E. miletus* from different regions were the Firmicutes, Bacteroidetes and Spirochaetes. The Chao1 index was used to assess the community’s richness, and the larger the value, the higher the species richness (Wang et al., 2020). A steady and nutrient-rich environment supplied by the host is also necessary for the survival and reproduction of gut microbes (Bäckhed et al., 2004). The gut microbiota of *E. miletus* in XGLL abundance declined following food restriction, according to α diversity analysis. This could be explained by the fact that there was less food available at higher altitudes, which led the organisms to respond by reducing the number of certain microorganisms in order to conserve energy. Higher Shannon index has been found to correlate with better bacterial community stabilization and increased disease resistance (Liu et al., 2023). The results of the food restriction treatment indicated that food reduction may result in a decrease in the stability of the bacterial community in the intestinal tract, which weakens their resistance to disease. Shannon index of *E. miletus* was lower in XGLL than that of in JD, that is contrary to previous studies on the intestinal microbiota of goats (Zhang et al., 2024). We speculated that this phenomenon occurs because of differences in physiology, digestive systems, and behavior between *E. miletus* and goats, which influence their reliance on and selection of gut microbiota. *E. miletus* may be more dependent on specific microbes to cope with high altitude environments, whereas goats’ digestive systems may be more inclined to promote microbial diversity. Moreover, there is a non-significant decreasing trend in Shannon’s index in *E. miletus* in both JD and XGLL.

The animals exhibit broader diversity of gut community as the altitude increases, which is consistent with the adaptation of rhesus monkey and plateau pika gut microbes to high-altitude environments (Li et al., 2018; Wu et al., 2020). A comparison of the JD and XGLL control groups revealed that the gut community richness of *E. miletus* was greater at higher altitudes. Animals that live at high altitudes have a variety of environmental obstacles, including low temperatures, reduced oxygen levels, and higher UV radiation (Liu, 2020). To adapt to these changes, their gut microbiota has undergone adaptive alterations. The animal gut microbiota is more adaptive due to the increased community diversity, and it can aid the host in surviving and thriving in the relatively harsh environment of high altitude. Important ecological traits of gut community include stability, resistance, and resilience (Lozupone et al., 2012). It showed that the *β* diversity of gut microbiota in *E. miletus* was not significantly affected by food restriction treatments or different regions, which was inconsistent with the results of previous studies of gut microbiota in wild pikas. Plateau pika (*Ochotona curzoniae*) is an important high-altitude model animal, the β-diversity of their gut microbiota increased with elevation (Li et al., 2019), but for *E. miletus*, their diet was relatively monotonous, indicating that the gut microbial community of *E. miletus* was relatively stable and resistant to some degree of external interference.

In the present study, there were less OTUs in XGLL than in JD. Rhesus monkeys and bumblebees both showed a decline in the OTUs of gut bacteria with an increase in altitude, reflecting that the gut community has adapted to the high-altitude environment (Wu et al., 2020; Zhang et al., 2023). We speculated that only microorganisms adapted to this more extreme environment may survive at high altitude, as a result of a natural selection pressure exerted by the comparatively harsh environment of high altitude on animal gut microbiota. In the JD and XGLL regions, there was little difference between endemic and shared microorganisms. Animal gut microbial communities are frequently greatly impacted by their host species (Wang et al., 2022). Research has demonstrated that at varying elevations, the variety and quantity of gut microbiota in striped strong-ribbed lizards are similar (Montoya-Ciriaco et al., 2020). There is minimal diversity in the gut microbial species of the same animal species since their physiological traits and metabolic processes are comparable throughout geographic regions. Furthermore, individual gut microbiota abundance is more stable over time, and the effects of environmental differences across different places may not be significant (Chen et al., 2021). Animal gut microbiota may therefore stay essentially unchanged in a variety of settings.

The genera of gut microbes enriched in *E. miletus* in JD and XGLL can be divided into Bacteroidetes and Firmicutes, as the enrichment analysis graph illustrated. In contrast to the Bacteroidetes in JD, the microorganisms in the control groups were richer in Firmicutes in XGLL due to its higher elevation, less plant growth, and food scarcity. Bacteriophages can break down proteins and carbohydrates, while Firmicutes can break down a greater variety of organic materials and fibers (Waite and Taylor, 2014; He et al., 2023). Because Firmicutes was physiologically well-adapted and breaks down fiber more forcefully, *E. miletus* may be more dependent on them for energy at higher elevations. Firmicutes, which contain genes involved in energy metabolism, showed a notable enrichment in the food restriction groups in both locations (Kaakoush, 2015), and Bacteroidetes, which breaks down polysaccharides (McKee et al., 2021). *E. miletus* may be altering their energy intake and metabolism mechanisms to gather energy more efficiently, as evidenced by the phenomenon’s occurrence after food limitation. Further, it can be shown that *AJDercreutzia* was enriched in XGLL but not in JD when comparing the genera enriched in the food restriction group in both locations. By producing isoflavones through metabolism, *AJDercreutzia* can have anti-inflammatory properties (Galipeau et al., 2021). Isoflavones possess anti-inflammatory, anti-oxidant, immunomodulatory, and antifibrotic qualities (Alipour and Karimi-Sales, 2020). Furthermore, it has the potential to mitigate irritable bowel syndrome and improve intestinal community (Huang et al., 2022). Thus, we speculated that in a high-altitude, food-limited environment, the gut microbiota of *E. miletus* experienced distinct adaptive modifications in response to potential inflammatory reactions.

### 4.3 Most of the gut microbes were significantly correlated with physiological indicators, and gut microbes were adapted to the high-altitude environment by enriching for fat metabolism-promoting and cellulose-degrading bacterial genera

Figure 11 demonstrated the significant and negative correlation between BAT mass and the presence of *Bacteroides* and *Ruminococcus*, as well as the strong and negative correlation between WAT mass and the abundance of *Turicibacter*. Small mammals’ BAT, which uses energy to generate heat (Ghesmati et al., 2024). Following a food restriction, BAT’s weight decreased in *E. miletus*. Because of its potent ability to use polysaccharides, *Bacteroides* can nourish other bacteria and promote the symbiotic relationship of the gut community (Rakoff-Nahoum et al., 2016). Reduced food intake brought on by an increase in altitude may result in an increase in *Bacteroides* abundance, which would facilitate the organism’s adaptation to a food shortfall. This will improve how well food sugars are absorbed and used. Studies have indicated that a notable proportion of rumen bacteria are called *Ruminococcus*, and that diets high in fiber often lead to an increase in *Ruminococcus* abundance (Morrison and Miron, 2000). Our results were consistent, and one possible reason was that when food intake was restricted at high altitude and the food’s fiber content was contained, *Ruminococcus* becomes more numerous, making better use of the limited nutritional resources. When one consumes less food, their body breaks down WAT to replenish lost energy from daily metabolism. WAT functions as an energy reserve, an endocrine organ that regulates whole-body metabolism, and a source of fatty acids for other tissues through lipolysis as needed (Qian et al., 2022). In rodent and human studies, the relative abundance of *Turicibacter* is usually negatively correlated with dietary fat (Lynch et al., 2023). The fact that *Turicibacter* abundance increased following the dietary restriction treatment is likewise in line with our findings. Thus, WAT mass showed a significant negative correlation with *Turicibacter* abundance.

The majority of the physiological parameters of *E. miletus* in XGLL corresponded with the predominant genus of gut microorganisms. A significant negative association was seen between the abundance of *Lactobacillus* and most physiological indices, such as body mass, liver weight, heart weight. *Lactobacillus* abundance was significantly increased in both animal and human obesity studies (Drissi et al., 2017), which is closely related to the physiological functions of *Lactobacillus*. *Lactobacillus* can affect the balance of the intestinal community (Walter, 2008), producing lactic acid and short chain fatty acids that lower the pH of the intestinal tract and regulating the adaptation of other bacteria (Slattery et al., 2019). These acids can aid in weight control by improving lipolysis and oxidation and reducing the formation and accumulation of fat. They can also regulate energy and fat metabolism. At low pH values and in bile, lactobacillus can survive (Lynch et al., 2023). Additionally, by promoting the release of host antimicrobial peptides, acetic acid and propionic acid, which are generated by metabolism, can function as antimicrobials (Fukuda et al., 2011), which helps to maintain intestinal health. Furthermore, by producing short-chain fatty acids, *Lactobacillus* can induce the body to release the tyrosyl peptide PYY and glucagon-like peptide 1 (GLP-1) (Psichas et al., 2015). PYY controls intestinal motility, improves satiety, and decreases food intake, which lowers body mass; SCFAs suppress appetite and energy intake by encouraging the synthesis and secretion of PYY and GLP-1 by intestinal epithelial gland cells; and GLP-1 increases insulin secretion and sensitivity in the body, which prevents stomach emptying and encourages intestinal peristalsis (Delzenne et al., 2005; Byrne et al., 2015; Chambers et al., 2015). In our study, it showed that there had a positive correlation between Tc and S24-7(UG) abundance. It has been demonstrated that diabetic-sensitive animals given a high-fat diet have an increase in S24-7 abundance (Serino et al., 2012). This supports our findings that the body speeds up lipid metabolism to provide energy for daily tasks when food intake is restricted. It also restricted the amount of dietary cholesterol that is available and the liver’s rate of synthesis. It has been proven that T*uricibacter* influences bile acids and lipids in the host, which can lower adipose tissue mass and serum cholesterol (Lynch et al., 2023). Following food limitation, blood levels of glu drop, and *Turicibacter* may supply energy by increasing the abundance of the bacteria that break down fat and cholesterol. Adipose tissue secretes the hormone leptin, and the amount of this hormone in the serum varies with the size of the animal’s adipose tissue (Friedman and Halaas, 1998). In adipose tissue, *Lactobacillus* stimulates oxidative phosphorylation, which increases energy expenditure (Yoon et al., 2020). Therefore, there was a negative correlation between leptin and *Lactobacillus* quantity.

It showed that *Treponema* and *Bacteroides*, the two predominant bacteria of *E. miletus* in XGLL, helped *E. miletus* adapt to the high-altitude habitat. It has been shown that *Treponema* is associated with cellulose degradation (Baniel et al., 2021). Cellulose, a major component of plant cell walls, was also frequently present in plant-based diets that *E. miletus* may eat. Therefore, when there was a shortage of food, *Treponema* may help the body use dietary resources more effectively for energy and nutrients by breaking down polysaccharides. It has been demonstrated that *Bacteroides* stimulate the bile acid-TGR5-PPAR-*α* axis to activate fat oxidation in adipose tissue (Yang et al., 2017). Tg and *Bacteroides* abundance showed a positive correlation, which may indicate that *Bacteroides* promoted fatty acid oxidative degradation, which increased the host body’s energy source and helps *E. miletus* survive and proliferate in high-altitude environments.

RAD analysis showed that the physiological parameters of *E. miletus* responded differently to the relative abundance of distinct dominating bacteria in its intestine. The interactions within the intestinal population of *E. miletus* could be the cause of the occurrence. Figure 14 demonstrated the positive and negative correlations, respectively, between the abundance of *Lactobacillus* and *Turicibacter* and BAT mass. BAT mass was an important effector tissue for adaptive thermogenesis in humans and rodents (Greenhill, 2024), and was also linked to energy metabolism and body mass regulation (Liu et al., 2020). T*uricibacter* can modify lipid and bile acid metabolism, which impacts host lipid metabolism (Lynch et al., 2023). Therefore, by affecting bile acid and lipid metabolism, *Turicibacter* may indirectly alter BAT, however, more research is required to identify the precise mechanisms and effects. In the gut, *Lactobacillus* is a probiotic that controls key elements of the microbial community (Shi et al., 2024; Mani-López et al., 2022). Hence, we postulated that *Lactobacillus* might affect host energy balance and lipid metabolism via altering the composition of the gut microbial community. Network analysis is frequently used to infer the microbiome under theories of symbiosis, parasitism, and competition (Weiss et al., 2016). The co-occurrence network analysis reveals that microbes mostly cooperate with one another to support the stability and well-being of the gut environment.

## 5 Conclusion

The present study revealed the complex changes in gut community and physiological characteristics triggered by changes in the amount of food fed after food restriction in *E. miletus* captured at different altitudes. In this study, we discovered that while gut microbiota in *E. miletus* facilitates adaptation to high altitude, dietary limitation alters the composition of gut microbiota. Gut microbes cooperated to regulate metabolism and immunity during food shortage at high altitude. Smaller body mass, higher RMR, longer cecum, larger BAT weight, and lower leptin level were seen in *E. miletus* that survived at high altitudes. Changes in gut microbiota and physiological characteristics affecting flora species interactions and energetic homeostasis were significant in the adaptation of *E. miletus* to high altitude food fluctuations, providing a theoretical basis for future research on the mechanisms of high-altitude animal adaptation to the environment. Moreover, there is a connection between gut microbiota and physiological functions, but further research is required as a result of the current study’s inability to determine the precise mechanisms of this interaction.

## Data Availability

The datasets presented in this study can be found in online repositories. The names of the repository/repositories and accession number(s) can be found at: https://www.ebi.ac.uk/ena, PRJEB63215; https://figshare.com/, doi.org/10.6084/m9.figshare.27063499.
